# The War upon the Great White Plague

**Published:** 1909-11

**Authors:** Irving Fisher

**Affiliations:** President of the Committee of One Hundred on National Health


### The War Upon the Great White Plague.

BY PROFESSOR IRVING FISHER, PRESIDENT OF THE COMMITTEE OF ONE HUNDRED ON NATIONAL HEALTH.

To those who in the last ten years have taken part in the fight, it gives a thrill of joy to note how rapidly the number of recruits is now increasing, and to see the foe retreating; but before victory can be won, millions more of our countrymen must enlist, and millions of dollars must be spent. We must learn to realize that our deadliest foes are not the great nations across the sea, but the tiny microbes that surround us. It is these microscopic enemies that destroy the major part of the human race, yet as Pasteur said: "It is within the power of man to rid himself of every parasitic disease." At the present time we are losing 150,000 lives a year from the "Great White Plague," and these lives flicker out after an average illness of three and a half years each. Could all these lives be saved, over a billion dollars would be added to our annual national dividend, the average life time would be two years longer, and national efficiency and happiness would be increased in a ratio which statistics can not measure.

Tuberclosis must be stamped out by a combination of both public and private hygiene. Public hygiene must include not only more sanatoria, dispensaries, visiting nurses, model tenements, sterilizer, pasteurizer, or best of all, clean milk, mitigation of factory dust, disinfection of infected houses, enforcement of anti-spitting regulations, cleaner streets, etc., but what is probably of greatest importance, though least emphasized, a widespread system of isolation homes for incurables, such as have been advocated in Connecticut by Dr. Foster. The statistics of Nedsholme show conclusively that the death rate from consumption declines in proportion as infectious consumptives are isolated.

Private hygiene is even more important, and means a revolution in our habits of living. It means fresh air perpetually flowing through our homes and more of our lives spent out doors. It means common sense in diet—the avoidance of bolting food, from which dyspepsia springs; and the re-education of normal food instincts, the avoidance of gluttony on the one side and body starvation on the other, the avoidance of alcohol, the most potent of the predisposing causes of tuberculosis, and the avoidance of dirty, infected milk and meat. It means the "simple life," free from over-exertion on the one hand and indolence on the other; the habit of normal sleep, and the emancipation from worry.

In giving this prescription, Dr. Trudeau once said to me: "It is as simple as bathing in the waters of Jordan, and that is why people are so slow to follow it."

But today people are following, and following rapidly. When they see a man, who a few years ago was so ill of tuberculosis that he could scarcely drag himself out upon a porch now run twenty-five miles for pure love of exercise, or when they see nine college men inside of half a year double their endurance through rational diet alone, or when they learn that ex-President Roosevelt developed from a weak, timid boy into the personification of strength and courage, and that Cornaro, about to die at thirty-seven, abjured all unhygienic habits and prolonged his life to one hundred and three, they begin to realize the practical value of personal hygiene.

This is an age dominated by ideals of work, not leisure; but our vices and unhygienic habits have been handed down from an age when leisure and even helplessness were fashionable. To be able to get drunk was once an object of ambition, for it indicated a command of leisure; but today, when power to work is the ambition of all classes, it must come about that habits which promote that power will be adopted. The ideal of fashion is becoming efficiency, not idleness; and striving for such an ideal will not only wipe out the "Great White Plague," but many other scourges as well.—*Journal of the Southern Medical Association.*

